# Lack of Correlation of Plasma HDL With Fecal Cholesterol and Plasma Cholesterol Efflux Capacity Suggests Importance of HDL Functionality in Attenuation of Atherosclerosis

**DOI:** 10.3389/fphys.2018.01222

**Published:** 2018-09-11

**Authors:** Neelam Srivastava, Angelo B. Cefalu, Maurizio Averna, Rai A. K. Srivastava

**Affiliations:** ^1^Department of Internal Medicine, University of Palermo, Palermo, Italy; ^2^Integrated Pharma Solutions, Philadelphia, PA, United States

**Keywords:** HDL, mouse, PPAR-α, LXR, reverse cholesterol transport, cholesterol efflux, ABCA1, atherosclerosis

## Abstract

A number of clinical findings suggested HDL-raising as a plausible approach to treat residual risk of CVD. However, lack of CVD risk reduction by elevated HDL cholesterol (HDL-C) through cholesterol ester transfer protein (CETP) inhibition and enhanced risk reduction in apolipoprotein A-I Milano (apoAI-M) individuals with low HDL-C shifted the focus from HDL-C level to HDL function. In the present study, we investigated correlations between HDL-C, HDL function, fecal cholesterol excretion, and *ex vivo* plasma cholesterol efflux capacity (CEC) in animal models using two HDL modulators, LXR and PPAR-α agonists. In C57Bl mice, LXR agonist, T1317, *raised* HDL-C by 30%, while PPAR-α agonist, fenofibrate, *reduced* HDL-C by 30%, but fecal cholesterol showed twofold increase in both cases. CEC showed a 30–40% increase. Combination of LXR and PPAR-α agonists showed no changes in HDL-C, but, interestingly, fecal cholesterol increased by 4.5-fold, and CEC by 40%, suggesting existence of additional pathway for fecal cholesterol excretion. Regression analysis showed a lack of correlation between HDL-C and fecal cholesterol and CEC, while fecal cholesterol showed significant correlation with CEC, a measure of HDL function. ABCA1 and G1, the two important players in RCT showed greater induction with LXR agonist than PPAR-α agonist. HDL-C increased by 40 and 80% in LXR and PPAR-α treated apoA-I transgenic mice, respectively, with 80% increase in fecal cholesterol. A fivefold increase in fecal cholesterol with no correlation with either plasma HDL-C or CEC following co-treatment with LXR and PPAR-α agonists suggested existence of an HDL-independent pathway for body cholesterol elimination. In hyperlipidemic diabetic ob/ob mice also combination of LXR and PPAR-α agonists showed marked increases in fecal cholesterol content (10–20-fold), while HDL-C rise was only 40%, further suggesting HDL-independent elimination of body cholesterol in mice treated with combination of LXR and PPAR-α agonists. Atherosclerosis attenuation by LXR and PPAR-α agonists in LDLr-deficient mice was associated with increased fecal cholesterol, but not HDL-C. However, fecal cholesterol counts showed inverse correlation with aortic cholesteryl ester content. These data suggest: (a) lack of correlation between HDL-C and fecal or aortic cholesterol content; (b) HDL function (CEC) correlated with fecal cholesterol content; (c) association of reduced aortic lipids in LDLr^−/−^ mice with increased fecal cholesterol, but not with HDL-C, and (d) existence of an HDL-independent pathway for fecal cholesterol excretion following co-treatment with LXR and PPAR-α agonists.

## Introduction

Coronary artery disease remains the leading cause of death in the United States and other developed countries (American Heart Association HDass-u, 2007). While elevated levels of LDL-C and triglycerides are established risk factors for developing CAD (Cannon et al., 2004), low levels of HDL is suggested to be a risk factor for developing premature atherosclerosis (Linsel-Nitschke and Tall, 2005), and may represent the residual risk factors not covered by statins (Gordon et al., 1986; Diabetes Atherosclerosis Intervention Study Investigators [DAIS], 2001). Although an inverse correlation between HDL level and the risk of CADs has been suggested (Frick et al., 1987; Rubins et al., 1999), recent efforts to raise HDL through CETP inhibition were disappointing (Barter et al., 2007; Schwartz et al., 2012). Low HDL-C levels are the most common lipid abnormalities observed in men with CAD (Genest et al., 1991). Metabolic Syndrome (MetS) is characterized by a clustering of risk factors leading to developing CVDs (Reaven, 1995; Srivastava and Srivastava, 2004; Moller and Kaufman, 2005). Low HDL-C is identified as one of the features of MetS. The most important atheroprotective function of HDL, however, is the HDL-mediated enhancement of RCT, a process in which HDL receives excess cholesterol from the peripheral tissues, including macrophages in the arterial wall, and is subsequently delivered to the liver for biliary excretion (Srivastava and Srivastava, 2000).

Since apoA-I has been shown to specifically bind to ATP binding cassette transporter protein A1 (ABCA1) (Oram et al., 2000), the lipid-poor apoA1 (preβ-HDL) functions as an acceptor of cholesterol and phospholipid in an ABCA1-dependent manner resulting the formation of mature cholesterol ester rich spherical α-HDL particles following the action of lecithin cholesterol acyl transferase (LCAT). Indeed, overexpression of ABCA1 in mouse macrophages enhanced cholesterol efflux (Wang et al., 2000) further supporting the key role of ABCA1 in apoA-I-dependent cholesterol efflux from cells. The importance of macrophage ABCA1 expression in atherosclerosis has been further demonstrated in bone marrow transplantation of ABCA1 null macrophages in hyperlipidemic mice (Aiello et al., 2002). Conversely, the over-expression of macrophage ABCA1 in mice reduces atherosclerotic lesion development in low-density lipoprotein receptor deficient mice (Van Eck et al., 2006).

Many factors influence the ability of HDL to efflux cellular cholesterol to acceptor apoproteins, including modification of the acceptor proteins under conditions of oxidative stress (Kresanov et al., 2013) and inflammation (Morgantini et al., 2011). Additionally, players in the cells that promote lipid efflux get influenced under certain pathophysiological states, including diabetes (Klein, 1995) and inflammation (Morgantini et al., 2011). In this study, we tested the hypothesis that circulating HDL may not always correlate with the HDL functionality and fecal cholesterol excretion. We used several animal models, including WT C57Bl and hyperlipidemic LDLr-deficient mice treated with two HDL modulating agents, LXR and PPAR-α agonists, either individually or combined. LXR-selective agonists are known to induce ABCA1 expression in cultured cells (Zanotti et al., 2008) and in animal models (Naik et al., 2006; Hazra et al., 2012). LXR-α agonists (Repa et al., 2000) and PPAR-α agonists induce the transcription of ABCA1 (Chawla et al., 2001; Chinetti et al., 2001). Addition of PPAR and LXR-α agonists showed additive effects on ABCA1 upregulation, suggesting that these agonists influence ABCA1 transcription via independent mechanism. Since LXRα was also induced by PPARα agonists, and since ABCA1 promoter harbors LXR element, it suggests that PPARα agonists induce ABCA1 gene expression via LXR-mediated pathway (Chinetti et al., 2001). Thus, LXR agonists increase HDL through upregulation of ABCA1. Since diabetic individuals are at greater risk of CVD, we also examined correlations between HDL concentrations and fecal cholesterol in diabetic ob/ob mice. Our findings suggest that there was lack of correlation between circulating HDL levels and cholesterol excretion, and that atherosclerosis burden was inversely associated with fecal cholesterol excretion than circulating HDL concentrations.

## Materials and Methods

### Animal Models

C57Bl (WT), apoA-I transgenic, LDLr^−/−^, ob/ob, and LDLr^−/−^ mice were used in this study. All animal studies were approved by the IACUC and all guidance were followed. Animals were obtained either from the Jackson Laboratories or bred in-house. Details of study procedures with each animal models are described below.

### Comparison of Isotopic and Non-isotopic Methods for Cholesterol Excretion in the Feces

**FIGURE 1:**
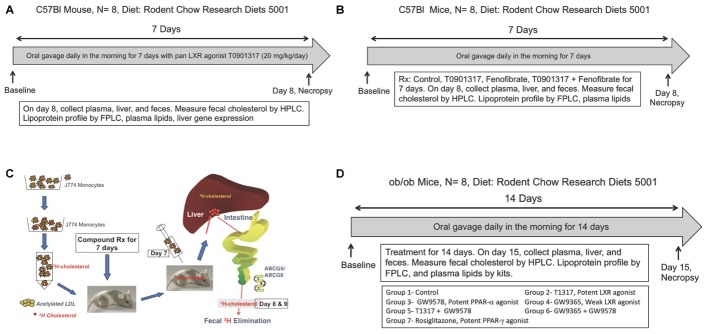
**(A)** Measurement of fecal cholesterol in the non-radioisotopic assay. C57Bl male mice were treated with pan LXR agonist, T1317, for 7 days followed by collection of feces over 24 h after last dosing and cholesterol content measured as described in Section “Materials and Methods.” **(B)** Quantitation of reverse cholesterol transport by measuring of macrophage-derived cholesterol in the feces. As shown, J774 cells were differentiated with acetylated LDL followed by loading radiolabeled cholesterol. C57Bl male mice (*n* = 8) were treated with pan LXR agonist, T1317, for 7 days. On day 6, after dosing, mice were administered intraperitoneally radiolabeled cholesterol loaded J774 cells and after 24 h, plasma, liver, and feces collected for [^3^H]-cholesterol counts. **(C)** Effect of LXR and PPAR-α agonists on fecal cholesterol excretion and plasma lipids. C57Bl mice (*n* = 8) were treated with pan LXR agonist, T1317, and PPAR-α agonist, fenofibrate, either individually or combined for 7 days followed by the measurement of fecal cholesterol and plasma lipids. **(D)** Effect of LXR and PPAR-α agonists on fecal cholesterol excretion and plasma lipids in male ob/ob mice. ob/ob mice (*n* = 8) were treated with pan LXR agonists, T1317 and GW3965, and PPAR-α agonist, fenofibrate and GW9578, either individually or combined for 14 days followed by the measurement of glucose, insulin, fecal cholesterol, and plasma lipids.

C57Bl male mice (10 weeks old, weighing 25–30 g) were used to carry out both radioisotopic and non-radioisotopic studies to compare fecal cholesterol excretion. A potent pan LXR agonist, T0901317 (T1317) (Crestani et al., 2004), was used as a reference agent for promoting ABCA1-mediated RCT (Zanotti et al., 2008) and using fecal cholesterol as a measure of RCT efficiency (Larrede et al., 2009). As shown in **Figure 1A**, for non-isotopic study, C57Bl mice were divided into two groups, one group (*n* = 8) was untreated control and the other group (*n* = 8) was administered potent pan LXR agonist, T1317, for 7 days by oral gavage once daily at a dose of 20 mg/kg/day. Mice were fed rodent chow from Research Diets (5001) *ad libitum* with free access to water. On the eighth day mice from both groups were sacrificed and plasma, liver and feces were isolated to measure plasma lipid profile, liver gene expression, and fecal cholesterol by HPLC (Homan and Anderson, 1998). Feces were collected only for the final 24 h during the treatment period. In terms of gene expression, blood monocytic ABCA1, G1 and hepatic SCD1, SREBP1, FAS mRNA were quantified.

In a second experiment relating to the validation of HDL concentration and RCT using two methods described above involved a dose–response experiment with a potent pan LXR agonist, T1317. In this experiment C57Bl mice were administered three doses of T1317 (0.3, 1.0, and 10 mg/kg/day) for 7 days. This was done using isotopic as well as non-isotopic method. Feces from both studies were collected 24 h after injection of radiolabeled cholesterol loaded macrophages. In the non-radioactive method, feces were collected after the sixth day of dosing over 24 h time period. In both studies biliary cholesterol was also measured by HPLC quantitation (non-radioactive study) or by counting radioisotope (radioisotopic study).

### Radioisotopic Method of RCT

*In vivo* radioisotopic macrophage-specific RCT was measured in J774 cell as described (Zhang et al., 2003; Naik et al., 2006). As illustrated in **Figure 1B**, J774 cells were grown, treated with acetylated LDL and then ^3^[H]-cholesterol was loaded (Zhang et al., 2003; Naik et al., 2006). First, mice were treated with the test agent for indicated time period followed by injection of radioisotopic cholesterol loaded J774 cells intraperitonially. After 24 and 48 h, feces, plasma, and liver, and in some studies bile, were isolated. Radioisotope was counted in all the fractions as described. LXR agonists are frequently used as a reference agent to study RCT in animal models (Naik et al., 2006; Hazra et al., 2012). We used a potent pan LXR agonist, T1317 (Zanotti et al., 2008). Groups of male C57Bl mice were treated with T1317 for 7 days. At the final day of dosing, plasma, liver, and feces were isolated and analyzed. Feces were collected over 24 h time period after the peritoneal injection of J774 cells treated with acetylated LDL and then loaded with ^3^[H]-cholesterol.

### Studies on HDL-C and RCT in C57Bl Mice

Male C57Bl mice 9–10 weeks old were procured from Jackson Laboratories, Bar Harbor, Maine and employed to investigate the functionality of HDL following treatment with pan LXR agonist, T1317, and PPAR-α agonist, fenofibrate, either individually or combined. LXR is known to elevate HDL-C (Attie et al., 2001; Hozoji-Inada et al., 2011) and up-regulate players in the RCT like ABCA1 (Donkin et al., 2010) and ABCG1 (Cummins and Mangelsdorf, 2006). PPAR-α agonist, fenofibrate, is also known to elevate HDL-C in humans (Arakawa et al., 2005) and increase fecal cholesterol content in mice (Mukherjee et al., 2008). T1317 was used at 10 mg/kg/day and fenofibrate at 100 mg/kg/day (**Figure 1C**). The combination treatment with LXR and PPAR-α agonists was done with 10 mg/kg/day of T1317 and 100 mg/kg/day of fenofibrate. The treatment was continued for 7 days with *n* = 8 in each group. On day 8, mice were fasted for 8 h and sacrificed to isolate plasma and feces for analysis.

### Studies on HDL-C and RCT in ApoA-I Transgenic Mice

As described above in Section “Studies on HDL-C and RCT in C57Bl Mice,” LXR and PPAR-α agonists were evaluated for their RCT efficacy using apoA-I transgenic mice. It should be noted that PPAR-α agonist, fenofibrate, while increases HDL-C in humans (Arakawa et al., 2005), it lowers HDL-C in mice because of differences in the regulation of mouse and human apoA-I gene expression (Vu-Dac et al., 1998; Srivastava et al., 2011). We therefore used apoA-I transgenic mice (Srivastava et al., 2000) expressing human apoA-I gene to investigate whether RCT and fecal cholesterol are driven by HDL-C concentration in mice and whether or not there is a correlation between HDL-C, HDL functionality as measured by *ex vivo* cholesterol efflux and fecal cholesterol content. Male apoA-I transgenic (apoA-I-Tg) mice were obtained from Jackson Laboratories, Bar Harbor, Maine. At the time of initiation of study, apoA-I-Tg mice were 9–10 weeks old. All the procedures followed were same as with C57Bl mice (**Figure 1C**).

### Studies on HDL-C and RCT in Hyperlipidemic and Diabetic ob/ob Mice

Since diabetic state has been shown to influence RCT (de Boer et al., 2012; Farbstein and Levy, 2012; Bao et al., 2015), we examined in diabetic ob/ob mice how HDL-C level correlates with fecal cholesterol content after treatment with LXR, PPAR-α, and PPAR-γ agonists. In this study, we included potent PPAR-α agonist and weak LXR agonist as well. Male ob/ob mice were obtained from Jackson Laboratories, Bar Harbor, Maine. These mice were 8 weeks old when received. After 1 week of acclimation, mice were bled retroorbitally and total cholesterol, triglycerides, glucose, and insulin measured. Based on these values mice were grouped (*n* = 6/group) such that the average values of each parameters were very similar. As shown in **Figure 1D**, ob/ob mice treated individually with potent LXR agonist, T1317, potent PPAR-α agonist, GW9578, weak LXR agonist, GW9365, and potent PPAR-γ agonist, rosiglitazone. Treatments with combination of potent LXR agonist plus potent PPAR-α agonist and weak LXR agonist plus potent PPAR-α agonist were also performed. The groups were as follows: Group 1- vehicle control; group 2- T1317, a pan potent LXR agonist (20 mg/kg/day); Group 3- GW3965, a weak LXR agonist (20 mg/kg/day); Group 4- GW9578, a potent PPAR-α agonist (10 mg/kg/day); Group 5- potent LXR agonist + potent PPAR-α agonist; Group 6- weak LXR agonist + potent PPAR-α agonist; Group 7- Rosiglitazone, potent PPAR-γ agonist (20 mg/kg/day). Mice were administered test agents by oral gavage in the afternoon right before dark cycle starts. The treatment was continued for 14 days. On day 15, mice were bled by cardiac puncture under anesthesia and liver was removed for mRNA quantitation. Feces were collected over 24 h after the 14th day dosing.

### Studies on RCT in C57Bl Mice Treated With LPS

Since inflammation has been suggested to impair RCT (McGillicuddy et al., 2009; Annema et al., 2010), effect of endotoxin-induced inflammation on plasma lipids and RCT was examined in WT male C57Bl mice. Mice (*n* = 8) were fed rodent chow diet for 1 week followed by intraperitoneal injection of lipopolysaccharide (0.5 mg/kg). One group was given only saline solution which served as untreated control. Six hours later ^3^[H]cholesterol loaded J774 macrophage were administered intraperitoneally. Another group (*n* = 8) was treated exactly the same way, but without the administration of ^3^[H]cholesterol loaded J774 macrophage. This group was used to measure plasma lipids and hepatic gene expression. After 24 h mice were sacrificed to isolate blood, feces and liver.

### Studies on RCT in Hyperlipidemic LDL Receptor-Deficient Mice

LDL receptor deficient mouse model has been widely used as an animal model of atherosclerosis to investigate the progression and regression as well as efficacy in attenuating arterial lipid deposition by antihyperlipidemic, anti-diabetic, and anti-inflammatory agents (Srivastava et al., 2006, 2015; Srivastava and He, 2010; Srivastava, 2011). Here, we investigated the role of RCT in hyperlipidemic LDLr-deficient mouse model in attenuating lipid deposition. Male LDLr-deficient mice (*n* = 12/group) were fed high fat diet (Srivastava et al., 2006) for 8 weeks followed by treatments with LXR agonist, T1317, at two doses (10 and 30 mg/kg/day) and PPAR-α agonist, GW9578 (10 mg/kg/day) for 6 weeks. One group was treated with only dosing vehicle without T1317. After 6 weeks of treatment mice in each group (*n* = 16) were divided into two groups, one group (*n* = 8) was sacrificed to measure plasma lipid profile and aortic lipid contents as described (Srivastava and He, 2010). The other group (*n* = 8) was used to measure macrophage-specific RCT by radio-isotopic method as described in Section “Radioisotopic Method of RCT” and **Figure 1B**.

### Measurements of Aortic Lipid Content

Aortic lipid contents were measured as described (Srivastava et al., 2006). Briefly, after the removal of blood, the vasculature was perfused first with cold PBS containing 5 mM EDTA through the left ventricle followed by carefully removing of all adventitious tissues connected to the aorta. The aorta from each mouse was removed from aortic root to the renal artery and placed in cold PBS for 15 s and taken out in another dish to remove excess solution by using a paper blot. Aortas were weighed individually and cut into small pieces followed by the extraction of lipids using chloroform/methanol (2:1) as described (Srivastava et al., 1991, 2006). The measurement of cholesterol ester was quantitated in the aortic lipid extract (Srivastava et al., 1991).

### Fecal Cholesterol Measurements

For non-radioactive fecal cholesterol transport assay, male mice (*n* = 8/group) were treated with the test agent for specified amount of days followed by collection of feces over 24 h period. The feces were extracted with solvent and cholesterol measured. Fecal cholesterol were measured by ELSD using a shortened method of Homan and Anderson (1998). In brief, 100 mg of each fecal sample was homogenized in 0.50 ml of 150 mM NaCl/5 mM MOPS/1 mM EDTA/0.01% PMSF followed by the addition of 0.5 ml of 150 mM NaCl 5 mM MOPS/1 mM EDTA and 200 μl of internal standard (2.0 mg/ml eicosanol dissolved in 2:1 MeCl_2_:MeOH). Samples were extracted with 2.0 ml of (2:1 MeCl_2_:MeOH) and quantified in Agilent Series 1100 HPLC system.

### Measurement of Plasma Cholesterol Efflux Capacity

To measure plasma cholesterol efflux capacity (CEC), first plasma apoB was depleted. Polyethylene glycol (40 μl), made by dissolving 20 g in 1000 ml of 200 mM glycine buffer, was added to 100 μl mouse plasma followed by incubation for 20 min at room temperature. The contents were centrifuged at 10,000 × *g* and 4°C for 30 min, and supernatant transferred to another clean Eppendorf tube as described (Rohatgi et al., 2014; Trieb et al., 2016). Cholesterol efflux capacity of apoB-depleted plasma was carried out as described (Rohatgi et al., 2014). In brief, J774 macrophages were grown in DMEM medium supplemented with 10% fetal bovine serum. To enhance uptake of [^3^H]-cholesterol, the cells were first incubated with 0.3 mM 8-(4-chlorophenylthio)-cyclic AMP in serum-free DMEM to induce ATP-binding cassette transporter protein (ABCA1). Labeling of cells with [^3^H]-cholesterol was done for 24 h and cells were washed once with DMEM. After washing the cells with phosphate-buffered saline, cells were incubated at 37°C with apoB-depleted plasma (2.5%) for 4 h. ApoA-I (20 μg/ml) was used as a control. After 4 h of incubation, the medium was removed and the cells in the well as a monolayer were washed and harvested. Radioactivity was measured in both medium and cells. Cholesterol efflux was expressed as radioactivity in the medium divided by the total radioactivity in the medium and the cells.

### Apolipoprotein A-1 Measurement

Mouse apolipoprotein A-1 concentrations in plasma samples were analyzed by Sandwich-ELISA kit procured from Elab Sciences (Catalog No. E-EL-M0130) according to the manufacturer’s instructions.

### Lipoprotein Profile and Plasma Lipid Measurements

Lipoprotein profile was measured as described before (Srivastava et al., 1997b, 2000). In brief, to determine the lipoprotein profile, pooled plasma (400 μl) from each group of mice were loaded onto two Superose 6 columns connected in series; 0.5 ml fractions were collected using a solution containing 0.15 mM NaCl, 0.02% sodium azide and 1 mM EDTA. In each eluted fraction, cholesterol was measured, and the fractions corresponding to VLDL-, LDL-, and HDL-C were pooled to measure respective lipoprotein concentrations. Plasma levels of total cholesterol and triglycerides were quantitated using commercial kits as described (Srivastava et al., 2011).

### Insulin and Glucose Quantitation

In the ob/ob mice, glucose and insulin were measured as described (Srivastava et al., 2006).

### Measurement of RNA by Quantitative PCR

Hepatic and blood monocytic mRNA were carried out by quantitative polymerase chain reaction following the established methods (Srivastava et al., 1999). Total RNA (50 ng) was reverse transcribed by reverse transcriptase and the resultant single stranded complimentary DNA was amplified using thermal cycler. The sequences of respective genes for mRNA quantitation were obtained from NCBI for designing primers.

### Statistical Analysis

Statistical analyses were performed by one-way ANOVA with the Dunnett’s multiple comparisons where applicable. Data were expressed as mean ± SD.

### Spearman Non-parametric Correlation Analysis

Spearman non-parametric correlation analysis was carried out by Prism 6 software to determine correlation and *p*-values using regression analysis. Prism 6 calculates exact *p*-values in regression analysis, which are shown in respective figures. Correlations were determined between apoA-I, HDL, cholesterol efflux, fecal cholesterol content, and aortic cholesterol content.

## Results

### LXR Agonist, T1317, Show Similar Fecal Cholesterol Excretion in Radioisotope and Non-radioisotope Assays

**FIGURE 2:**
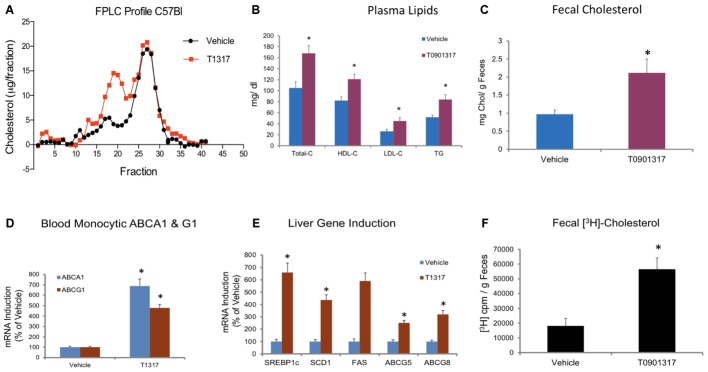
Male C57Bl mice (*n* = 8) were treated with pan LXR agonist, T1317, as described in **Figure 1C**. **(A)** lipoprotein profile; **(B)** Plasma lipids; and **(C)** fecal cholesterol. ^∗^ Significantly different (*p* < 0.01) compared to control; **(D)** ABCA1 and G1 expression in the blood monocytes; **(E)** Hepatic gene induction; and **(F)** fecal [^3^H]-cholesterol counts. ^∗^ Significantly different (*p* < 0.01) compared to control.

**FIGURE 3:**
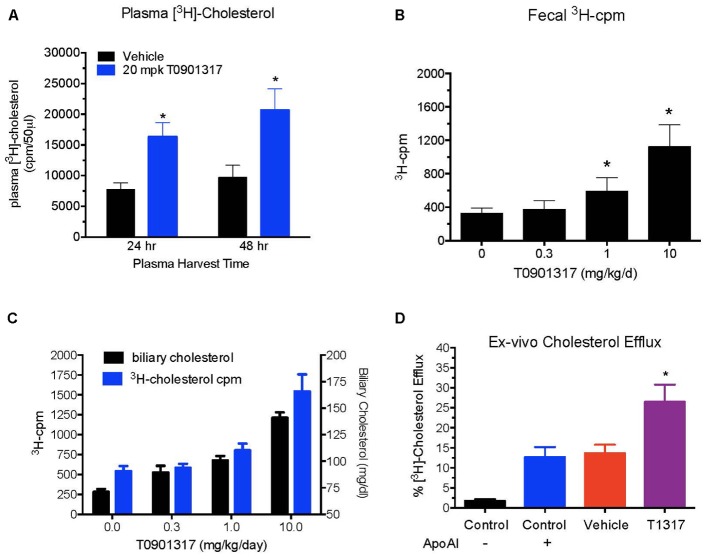
Dose–response of pan LXR agonist, T1317, on reverse cholesterol transport. Male C57Bl mice (*n* = 8) were treated as described in **Figure 2**, and reverse cholesterol measured as described in **Figure 1B**. **(A)** Macrophage-derived Plasma [^3^H]-cholesterol counts collected during 24 and 48 h after the last dosing of LXR agonist (20 mg/kg/day). **(B)** Dose–response of LXR agonist, T1317, on fecal [^3^H]-cholesterol counts. **(C)** [^3^H]-cholesterol counts and biliary cholesterol mass in bile. **(D)** *Ex vivo* cholesterol efflux in apoB-depleted serum. ^∗^ Significantly different (*p* < 0.01) compared to control.

To examine relationship between circulating HDL-C and HDL functionality measured by fecal cholesterol excretion as a maker of RCT, first we compared non-radioisotopic and radioisotopic method of RCT. As shown in **Figure 1A**, in non-radioisotopic method male C57Bl mice were treated with the reference agent, LXR pan agonist, T1317, known to elevate circulating HDL (Brunham et al., 2006) and enhances RCT (Zanotti et al., 2008) through induction of players (Delvecchio et al., 2008) in RCT pathway. Following 7 days of treatment, feces were collected, and total cholesterol measured. In the radio-isotopic assay, macrophage-specific RCT was measured. In this assay (**Figure 1B**), macrophages are loaded with radiolabeted cholesterol (^3^H) and after 7 days treatment with T1317, feces were collected and ^3^H counts were measured. The results of non-radioisotopic method of RCT measurement together with plasma lipid and hepatic RNA measurements are shown in **Figure 2**. As expected, the induction of lipogenesis (Schultz et al., 2000) and RCT (Zanotti et al., 2008) by LXR pan agonist, T1317 increased both LDL-C and HDL-C (**Figure 2A**). Compared to vehicle control, HDL-C, LDL-C, and TG increased in the LXR agonist treated mice (**Figure 2B**). Increased RCT as measured by fecal cholesterol (>2-fold) was evident in the T1317 treated mice (**Figure 2C**). As expected, increases in the fecal cholesterol in the T1317 treated mice paralleled ABCA1 and ABCG1 induction in blood monocytes (**Figure 2D**) and ABCA1, G1, G5, and G8 induction in the liver (**Figure 2E**). In the radioactive method of RCT measurement, T1317 showed increased RCT (**Figure 2F**) similar to the results in the non-radioisotopic method (**Figure 2C**). These results suggest that T1317 exhibited expected efficacy of increased fecal cholesterol in both the non-radioactive and radioactive methods. Twenty-four hour after the radiolabeled macrophage administration appears to be sufficient to show-up labeled cholesterol in the feces (**Figure 3A**). A dose-dependent increase in the fecal cholesterol was noticed in the T1317 treated mice (**Figure 3B**). Using both radioactive and non-radioactive methods, biliary cholesterol measurements showed similar dose-dependent increases (**Figure 3C**). These findings suggest that non-radioactive method provide similar results in terms of biliary cholesterol excretion as the radioactive method. Therefore, all subsequent studies examining HDL-C and RCT were carried out using non-radioisotopic method. However, in order to confirm that fecal cholesterol content indeed reflects HDL functionality, in a parallel experiment, *ex vivo* cholesterol efflux was measured in the apoB-depleted plasma and the results shown in **Figure 3D**, suggests that fecal cholesterol concentration in the T1317 treated mice also increased.

### Lack of Correlation Between HDL and RCT in C57Bl Mice

**FIGURE 4:**
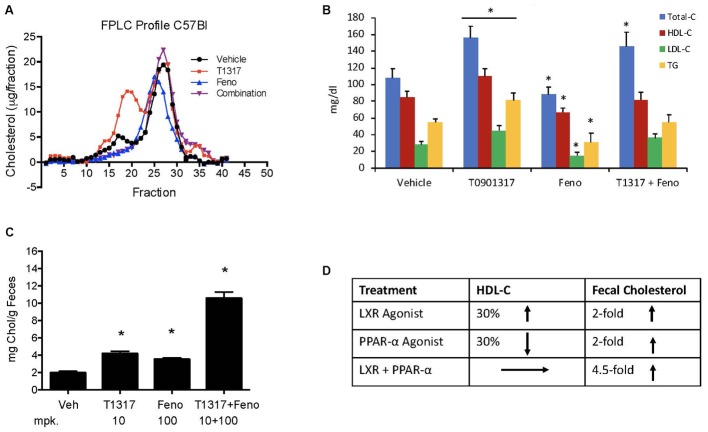
Effect of LXR and PPAR-α agonists on fecal cholesterol excretion and plasma lipids. C57Bl mice (*n* = 8) were treated with pan LXR agonist, T1317, and PPAR-α agonist, fenofibrate, either individually or combined for 7 days followed by the measurement of fecal cholesterol and plasma lipids. **(A)** Lipid Profile; **(B)** Plasma Lipids; **(C)** fecal cholesterol; and **(D)** cholesterol and plasma HDL-C. ^∗^ Significantly different (*p* < 0.01) compared to control. All data represent mean ± SD.

**FIGURE 5:**
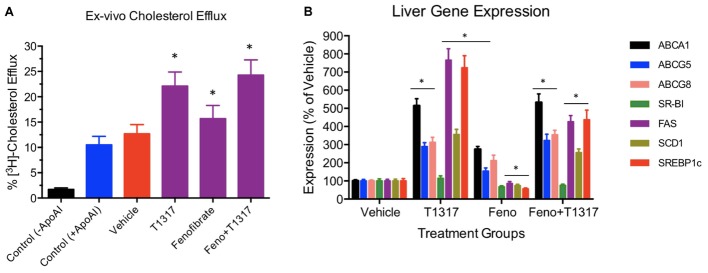
**(A)** *Ex vivo* cholesterol efflux assay to measure cholesterol efflux capacity (CEC) in the vehicle and treated mice plasma. Mouse plasma from vehicle and treatment groups were first processed to deplete apoB-containing particles. The remaining plasma was used to assay cholesterol efflux in J774 cells as described in Section “Materials and Methods.” For background efflux, media without apoA-I was used and for positive control 20 μg/ml apoA-I was used. ^∗^ Significantly different (*p* < 0.01) compared to control. All data represent mean ± SD. **(B)** Liver gene induction in the vehicle and treated C57Bl male mice. At the time of necropsy, 50 mg liver tissue was used to prepare total RNA and Q-PCR performed to measure mRNA. ^∗^ Significantly different (*p* < 0.01) compared to control. All data were presented as mean ± SD.

Correlation between HDL-C and RCT was examined using two reference agents, an LXR agonist that increases HDL-C in mice (Hozoji-Inada et al., 2011) and a PPAR-α agonist that decreases HDL-C in mice (Berthou et al., 1996; Vu-Dac et al., 1998), but increases in humans (Duez et al., 2005). As shown in **Figures 4A,B**, LXR agonist, T1317, known to increase HDL-C as well as hepatic lipogenesis through induction of a number of lipogenic genes (Schultz et al., 2000), increased HDL-C by 30% and fenofibrate decreased HDL-C by 30% (**Figures 4A,B**). Although fenofibrate reduced plasma levels of HDL-C, it resulted in the formation of larger HDL particles as evidenced by the shift of HDL peak toward left. This is consistent with the earlier findings that fenofibrate increases HDL size through induction of phospholipid transfer protein (PLTP) (Srivastava et al., 2011). However, fecal cholesterol excretion, a measure of RCT, was comparable in both LXR and PPAR-α agonist treated mice (**Figure 4C**). One group of mice was also treated with the combination of LXR and PPAR-α agonist, since the former increases lipogenesis, as evidenced by increased TG and LDL-C, and the later decreases lipogenesis (Srivastava et al., 2006) as evidenced by decreased TG and LDL-C (**Figures 4A,B**). Interestingly, while fecal cholesterol excretion increased by approximately twofold by LXR and PPAR-α agonists individually, a marked increase (4.5-fold) was noted in the fecal cholesterol excretion in mice treated with the combination of LXR and PPAR-α agonists (**Figures 4C,D**). It should be noted that there was no increase in the total circulating HDL-C in the combination group (**Figures 4A,B**). This necessitated direct measurements of the HDL functionality in a cholesterol efflux assay using the apoB-depleted plasma samples from all treated groups. As shown in **Figure 5A**, T1317 increased CEC parallel to the fecal cholesterol content and a somewhat similar trend was evident in the fenofibrate treated mice. However, despite 4.5-fold increase in the fecal cholesterol content, HDL function, measured as CEC, was comparable to T1317 treated group.

**FIGURE 6:**
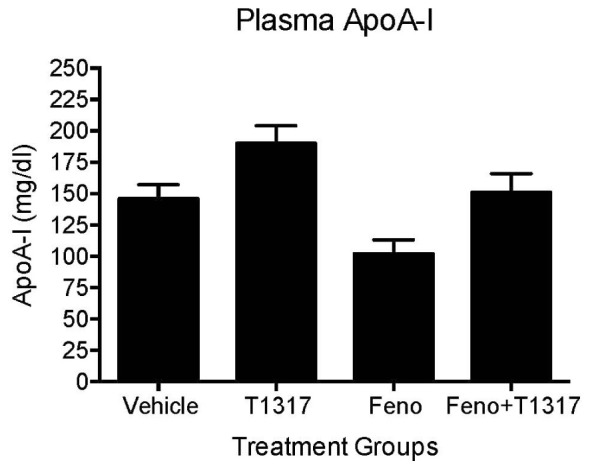
Mice were treated with vehicle, T1317, fenofibrate, and combination of T1317 and fenofibrate as described in **Figure 4**. At the end of 7 days treatment, individual mouse plasma was analyzed for apoA-I using commercial kit. Data shown are mean ± SD.

To further understand the role of players known to influence RCT and lipogenesis, expression of select genes in the liver were quantitated. As shown in **Figure 5B**, as expected, LXR agonist induced ABCA1, G1, G5, and G8 greater than the PPAR-α agonist and induced lipogenic genes, while PPAR-α agonist suppressed lipogenic genes, consistent with the reported findings (Schultz et al., 2000; Srivastava, 2009). The combination of LXR and PPAR-α agonist did not necessarily show additive effects on the RCT influencing genes, for instance ABCA1 and G1. SR-BI is a significant player in the uptake of HDL by the liver, which may influence fecal cholesterol content, however, in the current study LXR agonist did not significantly influence SR-BI, and fenofibrate suppressed SR-BI expression as reported earlier (Srivastava et al., 2011) ruling out the possibility of SR-BI in the fecal cholesterol content. Another important player in the RCT pathway is the apoA-I. We, therefore, measured plasma levels of apoA-I that functions as the acceptor of cellular cholesterol efflux in the ABCA-1 mediated pathway to find out if apoA-I levels explain some of the unexpected results on fecal cholesterol content in the combination treatment. LXR agonist does increase plasma apoA-I (**Figure 6**), which in turn raises HDL through ABCAI-mediated cholesterol efflux to apoA-I. Fenofibrate, on the other hand, reduces mouse apoA-I and HDL-C as reported earlier (Berthou et al., 1996; Vu-Dac et al., 1998). Combination of LXR and PPAR-α agonists showed no changes in the apoA-I or HDL-C as a result of positive (LXR) and negative (PPAR-α) effects on apoA-I gene expression.

**FIGURE 7:**
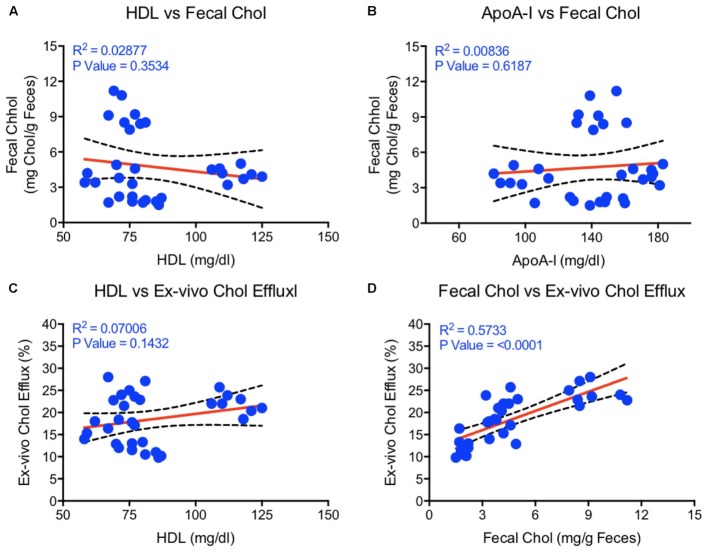
Linear regression analysis was carried out by non-parametric (Spearman) correlation using Prism 6 to determine correlations and *p*-values between HDL, apoA-I, fecal cholesterol, and *ex vivo* cholesterol efflux. **(A)** HDL vs. fecal cholesterol- no correlation; **(B)** ApoA-I vs. fecal cholesterol- no correlation; **(C)** HDL vs. *ex vivo* cholesterol efflux- no correlation; **(D)** Fecal cholesterol vs. *ex vivo* cholesterol efflux- highly significant correlation (*p* < 0.0001).

Correlation studies by regression analysis shown in **Figure 7** suggest that neither HDL-C (**Figure 7A**) nor apoA-I (**Figure 7B**) correlated with the fecal cholesterol content. Also, the HDL functionality measured as CEC did not correlate with HDL-C (**Figure 7C**). Interestingly, *ex vivo* cholesterol efflux (CEC), a measure of HDL functionality, showed significant correlation with the fecal cholesterol content (**Figure 7D**). In this correlation analysis, all data points were used, including the data on combination effects where HDL-C did not change, but fecal cholesterol increased by 4.5-fold. These findings confirm the validity of fecal cholesterol contents as a measure of RCT.

### Plasma Levels of HDL and RCT Does Not Show Correlation in ApoA-I-Tg Mice

**FIGURE 8:**
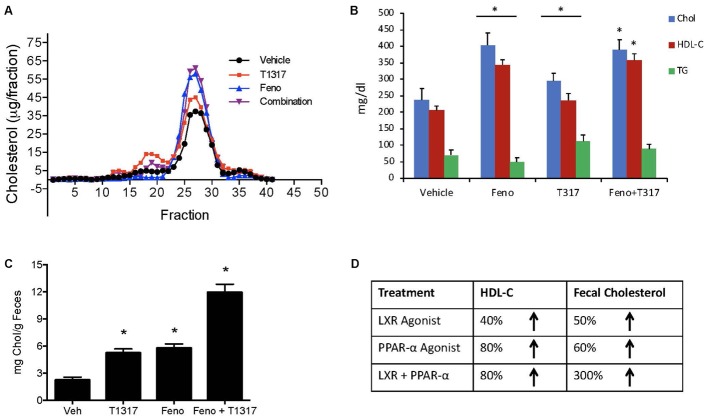
Effect of LXR and PPAR-α agonists on fecal cholesterol excretion and plasma lipids in apoA-I transgenic mice. ApoA-I Tg mice (*n* = 8) were treated with pan LXR agonist, T1317, and PPAR-α agonist, fenofibrate, either individually or combined for 7 days followed by the measurement of fecal cholesterol and plasma lipids. **(A)** Plasma lipoprotein profile; **(B)** Plasma lipids; **(C)** fecal cholesterol; and **(D)** comparison of fecal cholesterol and plasma HDL-C. ^∗^ Significantly different (*p* < 0.01) compared to control. Data presented show mean ± SD.

**FIGURE 9:**
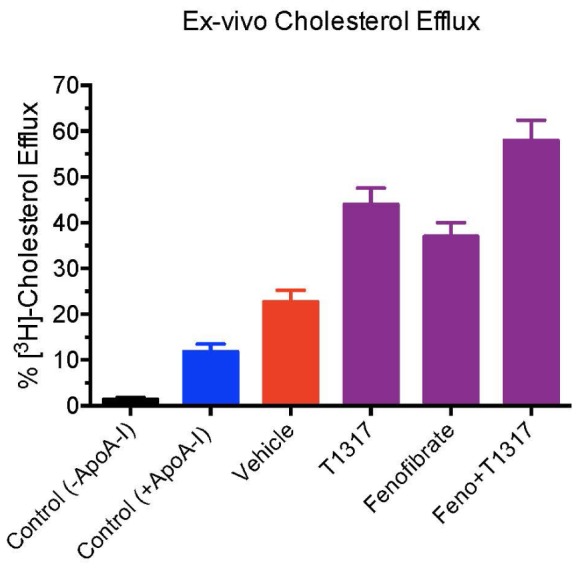
*Ex vivo* cholesterol efflux assay in apoA-I transgenic mice to measure cholesterol efflux capacity (CEC) in the vehicle and treated mice plasma. Mouse plasma from vehicle and treatment groups were first processed to deplete apoB-containing particles. The remaining plasma was used to assay cholesterol efflux in J774 cells as described in Section “Materials and Methods.” For background efflux, media without apoA-I was used and for positive control 20 μg/ml apoA-I was used. ^∗^ Significantly different (*p* < 0.01) compared to control. All data represent mean ± SD.

Fenofibrate, an PPAR-α agonist, although increases HDL-C in humans, it decreases HDL-C in mice as shown in **Figures 4A,B**. Therefore, we carried out same study with apoA-I Tg mice (Srivastava et al., 2000) following the same protocol as the WT C57Bl mice discussed above (**Figure 4**). In the apoA-I transgenic mice expressing human apoA-I, both LXR and PPAR-α agonists increased HDL-C. Although PPAR-α agonist showed twofold greater increases in HDL-C as compared to LXR agonist, T0901317, the fecal cholesterol increases were same (∼2-fold) when compared to untreated control (**Figures 8A,B**), suggesting a lack of correlation between HDL-C concentration and fecal cholesterol excretion. The combination of LXR and PPAR-α agonist treatment increased fecal cholesterol excretion by 5–6-fold (**Figure 8C**), although increases in HDL-C were same as the PPAR-α agonist treatment group (**Figure 8D**), suggesting a clear lack of correlation between HDL-C and fecal cholesterol excretion. To have mechanistic insights into the RCT pathway in apoAI-Tg mice, CEC was performed as a measure of HDL functionality. As shown in **Figure 9**, the basal CEC in the vehicle treated mice was twice that of non-transgenic C57Bl mice (**Figure 5A**).

### Discordance Between HDL Concentration and Fecal Cholesterol in Hyperlipidemic and Diabetic ob/ob Mice

**FIGURE 10:**
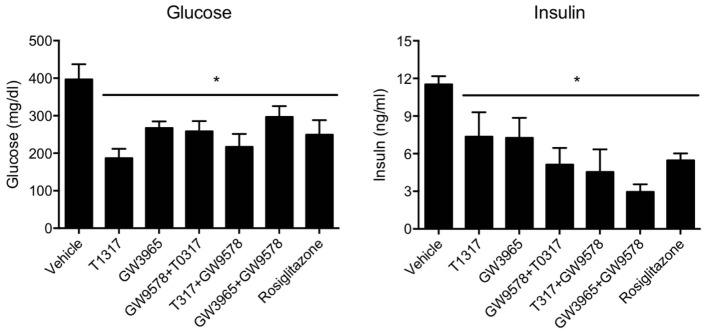
T1317, Potent LXR agonist; GW9578, Potent PPAR-α agonist; GW9365, Weak LXR agonist; Rosi- Rosiglitazone, Potent PPAR-γ agonist. Effect of LXR and PPAR-α agonists on plasma glucose (left panel) and insulin (right panel) in male ob/ob mice. ob/ob mice (*n* = 8) were treated with pan LXR agonists (T1317) and GW3965 (PPAR-α agonist), fenofibrate and GW9578, either individually or combined for 14 days followed by the measurement of glucose and insulin. ^∗^ Significantly different (*p* < 0.05) compared to control. Data presented are mean ± SD.

**FIGURE 11:**
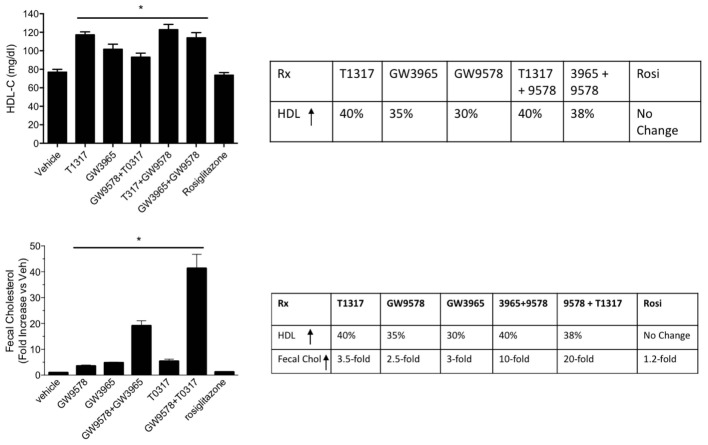
Effect of LXR and PPAR-α agonists on plasma HDL-C (upper panel) and fecal cholesterol (lower panel) in male ob/ob male mice. ob/ob mice (*n* = 8) were treated with pan LXR agonists, T1317 and GW3965, and PPAR-α agonist, fenofibrate and GW9578, either individually or combined for 14 days followed by the measurement of HDL-C and fecal cholesterol. ^∗^ Significantly different (*p* < 0.05) compared to control. Data are presented as mean ± SD.

Since diabetic conditions are associated with increased risk of CVD (Srivastava, 2018) and dampening of RCT, we examined HDL-C levels and fecal cholesterol in ob/ob mice treated with weak (GW3965) and potent (T1317) LXR agonists, respectively, potent PPAR-α agonist (GW9578), and potent PPAR-γ agonist (rosiglitazone). One group of mice was also treated with a weak LXR agonist (GW3965) plus GW9578, and one group with T1317 plus GW9578. Since PPAR-α, PPAR-γ, and LXR agonists have antidiabetic efficacy in animal model of diabetes (Srivastava, 2009), we first ascertained that these agonists show antidiabetic efficacy in ob/ob mice. As shown in **Figure 10**, all these agonists lowered glucose (left panel) and insulin (right panel). LXR agonist as well as combination of LXR and PPAR-α agonists showed maximal antidiabetic efficacy. Except PPAR-γ agonist, rosiglitazone, all agonists tested in this study increased HDL-C with greater HDL-C elevation in groups treated with T1317 and combination of LXR and PPAR-α agonists (**Figure 11**, upper panel). Fecal cholesterol measurements were done in all groups and results are shown in **Figure 11**, lower panel. Similar to the results shown above in WT C57Bl (**Figure 4**) and apoA-I Tg mice (**Figure 8**), T1317 showed 40% increase in HDL-C. In this hyperlipidemic diabetic model, potent PPAR-α agonist, GW9578, also showed 30% increase. Combination of LXR and PPAR-α agonist treatment did not further increase circulating HDL-C in ob/ob mice when compared to the treatment groups with LXR and PPAR-α agonist treatment individually. Most notably, potent PPAR-α agonist, GW9578 increased fecal cholesterol by 10-fold despite only 30% increase in HDL-C, when compared to untreated control. Combination of potent LXR and potent PPAR-α agonist treatment showed a massive 20-fold increase in fecal cholesterol, but only 38% increase in circulating HDL-C (**Figure 11**), suggesting role of HDL-independent pathway in body cholesterol excretion.

### Endotoxin-Induced Inflammation Reduces RCT in C57Bl WT Mice

**FIGURE 12:**
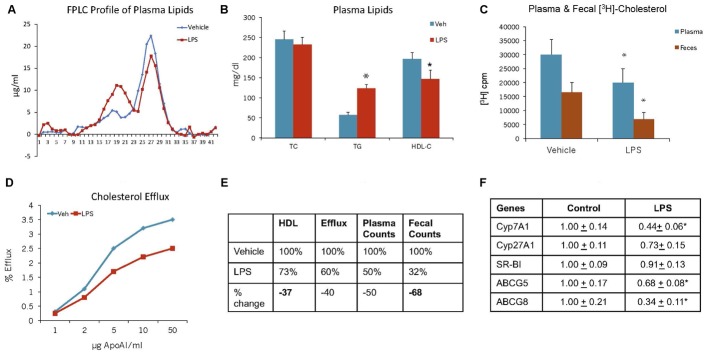
Mice were treated as described in Section “Materials and Methods.” **(A)** lipoprotein profile; **(B)** Plasma lipids; **(C)** Plasma and fecal [^3^H]-cholesterol counts; **(D)** *Ex vivo* cholesterol efflux of apoB-depleted plasma to varying concentration of apoA-I; **(E)** comparison of HDL-C and reverse cholesterol transport. ^∗^ Significantly different (*p* < 0.05) compared to control. **(F)** Hepatic mRNA measurements. mRNA expression levels were determined by real-time quantitative PCR in livers of C57BL/6J mice 48 h after the administration of either saline or LPS. Results are normalized to the expression of the housekeeping gene GAPDH and are expressed relative to the respective controls. ^∗^ Significantly different from saline controls (at least *p* < 0.05). Data presented as mean ± SD.

While HDL possesses anti-inflammatory properties (Navab et al., 2005; van der Westhuyzen et al., 2007), in addition to its primary function of RCT (Sacks and Jensen, 2018), under inflammatory conditions, HDL function gets compromised (McGillicuddy et al., 2009; Morgantini et al., 2011). We examined cholesterol efflux and reverse cholesterol capability of C57Bl mice under endotoxin-induced condition. The results shown in **Figure 12** suggest that pro-inflammatory conditions raise proatherogenic triglyceride-rich particles as evidenced by increased TG and VLDL (**Figure 12A**). Endotoxin-induced inflammation lowered HDL-C by 27% (**Figure 12B**). RCT measured by plasma and fecal cholesterol counts decreased by 50 and 64%, respectively (**Figure 12C**). *Ex vivo* cholesterol efflux also reduced (**Figure 12D**). Overall, HDL functionality, measured by fecal cholesterol counts, was compromised more than the HDL-C (**Figure 12E**). The dampened cholesterol efflux capability and RCT was corroborated by decreases in the expression of cholesterol 7-α hydroxylase A1 (Cyp7A1), ABCG5 and G8 (**Figure 12F**).

### HDL Functionality, but Not HDL-C, Correlates With Aortic Lipid Content in LDLr-Deficient Mice

**FIGURE 13:**
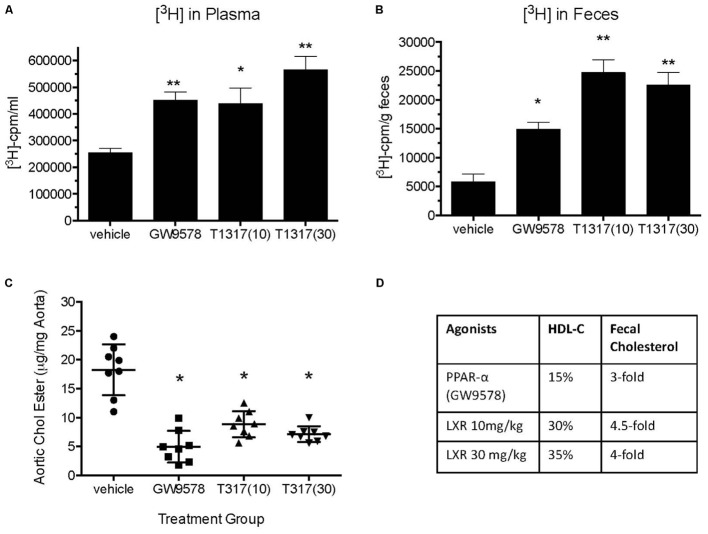
Effect of LXR and PPAR-α agonists on reverse cholesterol transport, HDL-C, and atherosclerosis burden in LDLr-deficient mice. Before starting treatment, male LDLr-deficient mice (*n* = 16/group) were fed high fat diet for 8 weeks followed by treatment as follows: Group 1, vehicle; Group 2, PPAR-α agonist GW9578 (10 mg/kg/day); Group 3, LXR agonist, T0901317 (10 mg/kg/day); Group 4, LXR agonist, T1317 (30 mg/kg/day). Mice were treated for 6 weeks. After 6 weeks of treatment mice in each group (*n* = 16) were divided into two groups (*n* = 8), one group (*n* = 8) was sacrificed to measure plasma lipid profile and aortic lipid contents as described. The other group was used to measure macrophage-specific reverse cholesterol transport by radio-isotopic method as described in Section “Materials and Methods.” **(A)** Plasma [^3^H]-cholesterol counts; **(B)** Fecal [^3^H]-cholesterol counts; **(C)** Aortic lipid content; and **(D)** comparison of plasma HDL-C, fecal [^3^H]-cholesterol counts, and aortic lipid content. ^∗^*p* < 0.01; ^∗∗^*p* < 0.001.

**FIGURE 14:**
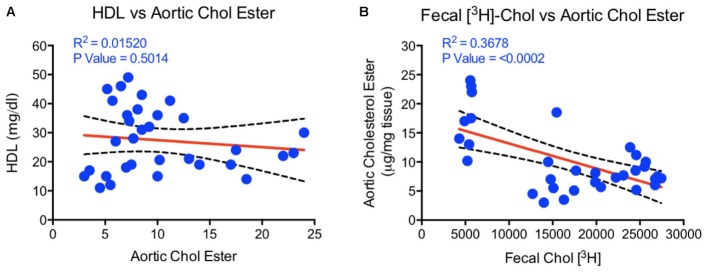
Linear regression analysis to determine correlations between HDL, fecal cholesterol counts, and aortic cholesterol ester content. **(A)** HDL vs. aortic cholesterol ester- no correlation; **(B)** aortic cholesterol ester vs. fecal ^3^[H]-cholesterol counts correlation- highly significant correlation (*p* < 0.0002).

To examine if aortic lipid deposition is influenced by plasma HDL-C, LDLr-deficient mice were fed-high fat diet and treated with HDL raising agents. LDLr-deficient model was used in this study because, with few exceptions, most mouse atherosclerosis models available are hyperlipidemia-induced models. While atherosclerosis progression in this model is apoB-containing proatherogenic lipoprotein driven (Srivastava et al., 2006), we asked the question if RCT and/or HDL-C is responsible for inhibition of progression in this model. As expected, both plasma and fecal ^3^H-cholesterol counts increased following treatments with PPAR-α and LXR agonists (**Figures 13A,B**). Fecal cholesterol excretion reached maximal level at 10 mg/kg/day dose of LXR agonist, T1317 (**Figure 13B**). There was a modest increase (15%) in circulating HDL-C following treatments with PPAR-α agonist, GW9578 (**Figure 13D**). Treatment with higher dose of LXR agonist, T1317, showed increased circulating HDL-C by 30%. To examine if fecal cholesterol excretion is any indication of aortic lipid content, we measured fecal cholesterol content in the feces and cholesteryl ester in the aorta. PPAR-α agonist, despite small increases in the circulating HDL-C, showed marked reductions in aortic cholesteryl ester content (**Figure 13C**). LXR agonist, T1317, also reduced aortic lipid content, but not as much as the PPAR-α agonist. A comparison of circulating HDL-C, fecal cholesterol count and aortic lipid contents shown in **Figure 13D** suggests inverse correlation between aortic lipid and fecal cholesterol counts in the PPAR-α agonist treated group to a great extent and in the LXR agonist-treated group to some extent. Thus, even in the LDL-driven atherosclerosis model, fecal cholesterol showed close association with aortic lipid lowering. A linear regression analysis was carried out to find out whether HDL-C and/or fecal cholesterol counts show correlation with aortic cholesteryl ester content (**Figure 14**). While plasma HDL-C did not show correlation with aortic lipid content (**Figure 14A**), fecal cholesterol counts showed significant inverse correlation with aortic lipid content (**Figure 14B**), demonstrating the importance of HDL functionality over HDL-C.

## Discussion

Low HDL-C as cardiovascular risk has been suggested since the first clinical study showing a reverse association between HDL-C and mortality (Gordon et al., 1989; Goldbourt et al., 1997). Considering the inverse correlation between HDL-C and CVD-related mortality, several approaches have been made to raise circulating levels of HDL-C, including CETP inhibition (Kosmas et al., 2016), LCAT activation (Chen et al., 2012), apoA-I induction (Di Bartolo et al., 2016), and infusion of apoA-I as unilamellar lipid vesicles (Balder et al., 2013). However, despite elevation of circulating HDL-C, the expected results were not obtained in outcome studies. These findings together with compromised HDL functionality in diabetics (Patel et al., 2011; Rohatgi et al., 2014), who are at 2–3-fold higher CVD risk compared to non-diabetic individuals suggest that factors beyond HDL-C may be involved in RCT. Indeed, higher oxidative stress (Jaleel et al., 2010) and inflammation (Okuda et al., 2012) as a result of elevated advanced glycation end products (AGEs) in diabetics dampens HDL functionality (Passarelli et al., 2005). Since CVD risk in diabetes correlated with *ex vivo* apoB-depleted plasma cholesterol efflux, we tested the hypothesis that HDL-C may not always correlate with RCT capability. We used several HDL-C elevating agents and animal models to test our hypothesis.

First, we optimized a non-radioisotopic assay to measure RCT capacity by determining fecal cholesterol content. Using a reference agent, LXR pan agonist, known to enhance RCT (Zanotti et al., 2008), we showed that non-isotopic assay compares well with the radioisotopic method, which specifically measures macrophage-to-feces transport of labeled cholesterol carried in circulation by macrophages (**Figure 1B**). As expected, the pan LXR agonist induced players in the RCT including ABCA1, G1, G5, and G8 (**Figure 2E**). LXR agonists are known to induce lipogenesis as well (Schultz et al., 2000), and in the present study, we did find induction of lipogenic genes, SREBP1c, FAS, and SCD1. Therefore, to avoid use of radioactive material, we preferred to use fecal cholesterol concentration as a measure of RCT in most of the studies reported here.

Fenofibrate (PPAR-α agonist) and T1317 are widely studied reference agents to investigate lipid regulation (Schultz et al., 2000; Srivastava et al., 2011) and atherosclerosis (Srivastava, 2011). We used these two reference agents to investigate a correlation between circulating HDL-C and fecal cholesterol excretion as a measure of RCT. Unlike in humans, fenofibrate lowers circulating HDL-C in mice because of the differences in the human and mouse apoA-I promoter (Vu-Dac et al., 1998). ApoA-I, the main protein component of HDL-C and a good acceptor in the process of cellular cholesterol efflux determines the circulating levels of HDL (Srivastava and Srivastava, 2000). In the C57Bl mice, as expected, fenofibrate lowered HDL-C by 30% and LXR agonist increased HDL-C level by 30%. Interestingly, fecal cholesterol increased by twofold in both cases regardless of whether HDL-C decreased or increased (**Figure 4**), suggesting that circulating HDL-C did not correlate with the fecal cholesterol content. To confirm that indeed fecal cholesterol content serves as a measure of RCT, *ex vivo* cholesterol efflux capacity (CEC) was measured, which paralleled fecal cholesterol content at least in mice treated with LXR and PPAR-α agonists individually. Combination treatment with PPAR-α and pan LXR agonist did not change circulating HDL-C, yet increased fecal cholesterol content by 4.5-fold, suggesting a lack of correlation between circulating levels of HDL-C and fecal cholesterol content. These finding indicates existence of HDL-independent non-biliary cholesterol elimination in the feces (van der Velde et al., 2007). Indeed, similar to present findings, HDL-independent TICE responsible for fecal cholesterol elimination has been described (Vrins et al., 2012). However, in the present investigation, we did not focus on the HDL-independent mechanism of fecal cholesterol excretion. To validate the fecal cholesterol content as a measure of HDL functionality, a correlation analysis was performed, and the results clearly demonstrated that HDL-C did not correlate with fecal cholesterol content, while *ex vivo* cholesterol efflux capacity (CEC) correlated significantly with the fecal cholesterol content (**Figure 7**). As shown in **Figure 4A**, the characteristics of PPAR-α and LXR agonists was evident by the marked reduction of TG following treatment with PPAR-α agonist (Srivastava et al., 2006) and increased TG by LXR agonist (Schultz et al., 2000). This confirms that the findings on HDL-C and RCT by these two agonists were mediated via established players in the RCT pathway (**Figure 5B**).

**FIGURE 15:**
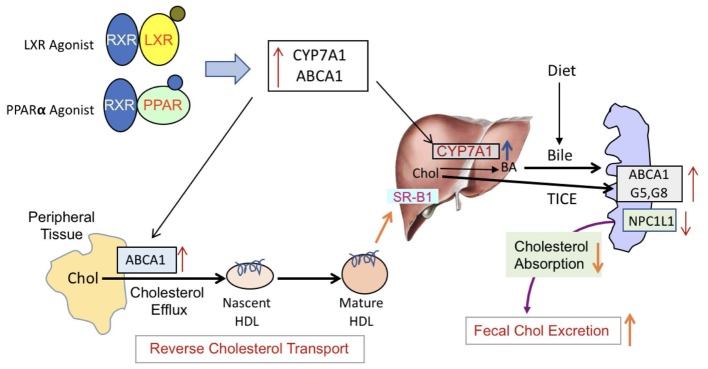
PPAR-α and LXR-mediated induction of RCT. LXR and PPAR-α agonists form a heterodimeric complex with RXR and induce respective responsive genes. LXR agonists are known to activate ABCA1, G1, G5, and G8 as well as Cyp7A1. LXR agonists also down-regulate NPC1L1 gene involved min cholesterol absorption and transintestinal cholesterol efflux. PPAR-α agonists also activate many of the same genes, albeit to a lesser extent. The major difference in LXR and PPAR-α agonists is that PPAR-α agonists inhibit lipogenesis while LXR agonists induce hepatic lipogenesis. Induction of ABCA1 promotes cellular cholesterol efflux to the lipid-poor nascent HDL particles and becomes mature HD particle, which are then taken up by the liver via SR-B1-mediated pathway. Cholesterol brought to the liver are converted to bile by Cyp7A1 which are elevated in the presence of LXR or PPAR-α agonists. Additionally, the induction of ABC transporter proteins as well as inhibition of NPC1L1in the gut induces TICE. These two major pathways, biliary and non-biliary, contribute to the fecal cholesterol excretion. In the presence of both the LXR and the PPAR-α agonists, these pathways are highly activated to result into higher cholesterol elimination in the feces.

To address the opposing effects on HDL-C by PPAR-α in rodent and humans (Vu-Dac et al., 1998) and how it influences RCT, we utilized transgenic mouse model overexpressing human apoA-I and carried out exactly the same experiment as with C57Bl mice. In apoA-I transgenic mice overexpressing human apoA-I, both PPAR-α as well as LXR agonists raised circulating HDL-C by 80 and 40%, respectively, while fecal cholesterol increased by 60 and 50%, respectively. Greater HDL-C increases in the PPAR-α-treated group was due to the upregulation of human apoA-I gene (Srivastava et al., 2011). Combining PPAR-α and LXR agonists did not further increase circulating HDL-C, but showed marked elevation (300%) in the fecal cholesterol content, once again suggesting the absence of direct correlation between HDL level and fecal cholesterol content. Thus, studies performed in wild-type C57Bl and apoA-I transgenic mice show agreement in terms of dissociation between circulating HDL-C and fecal cholesterol. It is possible that the fecal cholesterol contents may be the result of a combination of more than one physiological phenomenon, including LDL receptor-mediated uptake of LDL-C by the liver leading to the conversion into bile acids for excretion (Srivastava and Srivastava, 2000). Since mice have very low levels of circulating LDL-C on normal rodent chow (Srivastava et al., 1993), it appears unlikely that the majority of fecal cholesterol are derived from LDL receptor-mediated uptake of LDL-C by the liver. Other prominent players that influence RCT are SR-BI (Van Eck et al., 2005), ABCA1 (Joyce et al., 2002; Srivastava, 2002), and G1 (Kennedy et al., 2005). The composition of HDL may also influence RCT since pre-β HDL particles (Kane and Malloy, 2012) are implicated in the induction of RCT (Srivastava, 2002). In the present study, we have not addressed this mechanistic aspect since the goal of this study was to investigate plasma levels of HDL-C and RCT as measured by fecal cholesterol or fecal [^3^H]-cholesterol counts, and *ex vivo* cholesterol efflux. It is unlikely that RCT is positively influenced by SR-BI in the PPAR-α agonist treated group, since fenofibrate down-regulates SR-BI in the liver (Mardones et al., 2003; Srivastava et al., 2011). In addition, fenofibrate induces SR-BI degradation (Lan and Silver, 2005), ruling out the role of SR-BI in fecal cholesterol excretion. The PPAR-α agonist-mediated induction of SR-BI in macrophage may partly contribute to enhance RCT (Chinetti et al., 2000). Since PPAR-α agonist also influences prominent players in RCT via inducing transcription factor LXR selectively in the macrophages (Nakaya et al., 2011), this may induce ABC transporter proteins. The synergistic effects observed in the PPAR-α and LXR agonist combination treatment appears to occur partly as a result of induction of RCT pathway and also via HDL-independent pathway, including TICE (**Figure 15**), but this appears to be limited to the synergistic effects in the combination treatment group, since hepatic ABC transporter proteins are insufficient to explain massive increases in the fecal cholesterol content. LXR agonists are known to induce intestinal ABCG5 and G8 (Repa et al., 2002), which inhibits cholesterol absorption and induces TICE (van der Veen et al., 2009). It is also suggested that biliary cholesterol secretion may not be required for macrophage RCT (Temel et al., 2010). As shown in **Figure 15**, part of the fecal cholesterol in the combination of PPAR-α plus LXR agonists treated group could account from HDL-independent non-biliary mechanism involving other players like NPC1L1 that influence cholesterol absorption (van der Veen et al., 2005). Acyl CoA acetyl transferase-2 (ACAT2) is also known to influence cholesterol absorption in the gut (Temel et al., 2005). Since both PPAR-α (Valasek et al., 2007) and LXR (Duval et al., 2006) agonists down-regulate NPC1L1 gene, some of the effects seen on fecal cholesterol excretion may be mediated via NPC1L1 in mice treated with combination of PPAR-α and LXR agonists. Indeed, NPC1L1inhibitor, ezetimibe, enhances fecal sterol elimination through TICE-mediated pathway (Jakulj et al., 2016).

Given that the *ex vivo* cholesterol efflux correlated with CVD mortality in human clinical studies (Patel et al., 2011; Rohatgi et al., 2014; Traldi et al., 2015), one of the questions we asked in this study was to examine association between RCT and aortic lipid deposition. To test this hypothesis, we employed LDLr-deficient mice widely studied to evaluate progression of atherosclerosis (Srivastava et al., 2006) as well as to understand the biology of aortic lipid deposition (van Leeuwen et al., 2008). While this animal model of atherosclerosis is heavily driven by circulating LDL on high fat high cholesterol diet because of the absence of LDL receptor, nevertheless, we attempted to investigate the RCT component in the inhibition of atherosclerosis progression following treatment with LXR and PPAR-α agonists. In the absence of LDL receptor, another receptor, LRP1 carries out hepatic uptake of apoB/E-containing lipoproteins (Gordts et al., 2009). Fecal [^3^H]-cholesterol counts showed 3- and 4.5-fold increases in the PPAR-α and LXR treated animals, which appeared to correlate with the inhibition of aortic lipid deposition. However, there were only 15 and 30% increase in HDL-C in the PPAR-α (GW9578) and LXR (T1317) treated groups. *Ex vivo* cholesterol efflux did show induction in all treatment groups and an inverse correlation between fecal cholesterol counts and aortic cholesteryl ester content. Aortic cholesteryl ester showed a lack of correlation with HDL-C (**Figure 14**). Thus, present results clearly demonstrate that HDL functionality, measured as fecal cholesterol count or content correlated better with atherosclerotic lesion severity compared to HDL-C.

To look further into the details and to understand RCT in hyperlipidemic models, ob/ob mice was employed. All the studies discussed above used two reference compounds, fenofibrate (PPAR-α agonist) and T1317 (pan LXR agonist). Fenofibrate is a mild PPAR-α agonist and T1317 is a potent LXR agonist. To examine HDL-C and RCT correlation in ob/ob mice, we also used potent PPAR-α agonist (GW9578) and weak LXR agonist (GW3965) either individually or a combination of mild (GW3965) and potent (T1317) LXR agonists with potent PPAR-α (GW9578) agonist. In all the treatment groups, individually or combined, HDL-C increased in the range of 30–40%, but fecal cholesterol excretion increased in the range of 2.5- to 20-fold. The increases in the fecal cholesterol were related to the potency of the respective agonists, suggesting that induction of the RCT pathway, in part, played major roles compared to merely increasing the circulating HDL-C. One of the limitations of lack of increases in circulating HDL-C appears to be availability of the main protein component, apoA-I. It is possible that despite small or no changes in the circulating HDL-C, the RCT pathway may still be very efficient in delivering HDL-derived cholesterol to the liver and recycling apoA-I to serve as an acceptor for cellular cholesterol efflux. In this context macrophage ABCA1 and G1 are likely to play greater role in RCT as suggested (Aiello et al., 2002; Van Eck et al., 2006). More than additive effect on fecal cholesterol elimination in the combination treatment group with potent LXR agonist and potent PPAR-α agonist may have resulted from greater induction of ABCA1 and G1 in the macrophage on the one hand to promote cellular cholesterol efflux to nascent HDL particle that delivers it to liver for excretion, and induction of ABCG5 and 8 as well as inhibition of NPC1L1 by LXR and PPAR-α agonists (Duval et al., 2006; Valasek et al., 2007) on the other hand. The later possibly induced HDL-independent pathway to increase fecal cholesterol excretion (**Figure 15**).

In summary, the data presented using a number of animal models demonstrate that measurement of HDL functionality is more meaningful compared to measuring HDL-C, since even in cases where there was no change in HDL, CEC showed marked increases. These results further support clinical findings shown in individuals susceptible to CVD (Rohatgi et al., 2014; Traldi et al., 2015).

It should be noted that mice lack CETP and most of the cholesterol in mice are transported as HDL particle with low levels of circulating LDL (Srivastava et al., 1991). This results into fivefold lower plasma apoB in mice (Srivastava et al., 1997a) compared to humans (Pepin et al., 1991) and higher apoAI (Srivastava et al., 1992) compared to humans (Ghiselli et al., 1985). Mice have significantly lower biliary cholesterol secretion relative to biliary salt when compared to humans (van der Wulp et al., 2013). Therefore, transgenic mice expressing apoB and CETP may provide further insights into the physiology of RCT, biliary secretion, and TICE. Despite these limitations with mouse models, it presents relatively better genetic homogeneity and offers a variety of genetically modified strains to ask important biologic questions. In the present studies with mice, we believe that our findings add further understanding to the roles of HDL, RCT and fecal cholesterol elimination in attenuating atherosclerosis progression.

## Author Contributions

RS evaluated data from the studies and has contributed to the interpretation and analyses of data as well as writing of the manuscript. NS and AC designed the studies, carried out some of the experiments, and wrote part of the manuscript. MA contributed to the review and interpretation of data and wrote part of the manuscript.

## Conflict of Interest Statement

RS worked at Integrated Pharma Solutions and currently employed at Gemphire Therapeutics Inc. The remaining authors declare that the research was conducted in the absence of any commercial or financial relationships that could be construed as a potential conflict of interest.

## Abbreviations

ABCA: ATP binding cassette transporter
ACAT: Aacetyl-CoA acetyltransferase
CAD: coronary artery disease
CVD: cardiovascular disease
Cyp7A1: cholesterol 7-alpha hydroxylase
ELSD: evaporative light scattering detector
FAS: fatty acid synthase
HDL-C: high density lipoprotein cholesterol
LDL-C: low-density lipoprotein cholesterol
LRP1: LDL receptor related protein 1
LXR: liver x receptor
NPC1L1: Niemann-Pick C1-Like 1
PPAR: peroxisome proliferator activated receptor
RCT: reverse cholesterol transport
RXR: Rretinoid x receptor
SCD1: steroyl coA desaturase
SR-BI: scavenger receptor class B type 1
SREBP1: sterol response element binding protein 1
T1317: T0901317
TICE: transintestinal cholesterol efflux.
